# Multi-functional log-periodic graphene antennas for ultra-wideband systems

**DOI:** 10.1186/s11671-026-04447-3

**Published:** 2026-01-27

**Authors:** V. Govindaraj, C. Ramesh, S. Dhanasekar, Binay Kumar Pandey, Digvijay Pandey, Mesfin Esayas Lelisho

**Affiliations:** 1 Department of ECE, Kalaignarkarunanidhi Institute of Technology, Coimbatore, 641402 India; 2 Department of ECE, Sri Eshwar College of Engineering, Coimbatore, 641202 India; 3 Department of Information Technology, College of Technology, Govind Ballabh Pant University of Agriculture and Technology Pantnagar, Udham, Singh Nagar India; 4 Department of Technical Education, Lucknow, Uttar Pradesh India; 5 Department of Statistics, College of Natural and Computational Science, Mizan-Tepi University, Tepi, Ethiopia

**Keywords:** Metamaterial, Log periodic antenna, Ultra-Wide band, Sawtooth planar antenna, Plasmon

## Abstract

Ultra-wideband (UWB) technology has garnered significant interest from researchers worldwide. Reducing antenna size, guaranteeing radiation stability, attaining impedance matching, and keeping costs down are recent challenges.The adaptability, compactness, and wideband performance of log-periodic sawtooth planar antennas for UWB applications show great potential. There is much potential for improvement, especially in high-gain and multi-band settings, as indicated by the current research and advancements in resonator structures, reconfigurability, and meta surface designs. These advancements guarantee that log-periodic architectures will remain relevant and it targets wideband sub-GHz applications that complement 5G networks, including control, broadcast, and backward-compatibility services. A toothed log-periodic antenna based on graphene is proposed in this study to operate in the 0.1–1.3 GHz frequency range. In proposed method by varying the DC voltage applied to the graphene, the antenna’s bandwidth, radiation pattern, and operating frequency range is dynamically adjusted. The graphene’s chemical potential, surface conductivity, and surface impedance, this voltage control makes it possible to create a reconfigurable antenna design is adjusted. A log-periodic graphene lattice coupled to a 50-ohm feed line makes up the antenna’s radiating element. Simulation and implementation results demonstrate that the antenna generates stable, directional radiation patterns over a wide frequency range from 0.1 to 1.3 GHz when the chemical potential of graphene is set to 1 eV.

## Introduction

Log-periodic antenna is perfect for wideband applications such as UHF Ultra-Wideband (UWB). UHF provides a range of communication systems, including satellite and mobile, and operates between 300 MHz and 1.3 GHz. A variation on the log-periodic design, the planar sawtooth structure helps achieve excellent directivity and broad frequency coverage, which are essential for contemporary wireless systems. The sawtooth shape provides improved bandwidth and compactness, while the log-periodic design enables the wideband operation through variable element lengths. A Metamaterial-based Log-Periodic Antenna (LPA) for Ultra-Wideband (UWB) applications represents a significant advancement in antenna technology, leveraging the unique properties of metamaterials to achieve enhanced performance over a wide range of frequencies. UWB technology, with its ability to cover a broad frequency spectrum, is essential in various modern applications, including high-speed wireless communication, radar, and imaging systems. Traditional UWB antennas often face challenges such as bulky size, limited gain, and less control over beam direction. The integration of metamaterials—engineered materials with properties not typically found in nature enables unprecedented control over electromagnetic waves. By incorporating these materials into an LPA, it is possible to enhance antenna performance metrics like bandwidth, gain, and directivity, while also reducing the antenna’s physical size. The log-periodic design, known for its self-similar structure, offers natural frequency scalability and a consistent impedance profile across wide bandwidths, making it a popular choice for UWB applications.Metamaterial-based LPAs specifically cater to the needs of UWB applications by achieving high radiation efficiency and stable radiation patterns across multiple frequencies. This antenna design is especially suitable for environments where compact size, low mutual coupling, and high signal fidelity are required, such as in portable electronic devices, automotive systems, and medical equipment.A metamaterial-based log-periodic antenna for UWB applications provides an efficient, compact, and versatile solution that meets the demands of modern wireless and imaging systems, paving the way for innovations in high-data-rate, interference-resilient communication.

## Previous works

A small, planar UWB antenna with a wide impedance bandwidth that may operate at frequencies between 2.95 and 15.44 GHz was proposed by Azim et al. [1]. The design showed a flat, omnidirectional radiation pattern, which is essential for UWB communication systems. It had a slot structure and a partial ground plane. In a similar vein, Ziani et al. [2]. reported good efficiency (89%) and a broad bandwidth between 3.05 GHz and 13.98 GHz after optimizing a small square planar antenna. In order to improve gain and radiation properties and make their device appropriate for microwave imaging applications, they also incorporated a sawtooth shape. Bikrat et al. [3] developed a reconfigurable UWB planar antenna with PINdiode for interference suppression. This was further developed by Sahoo et al. [4], who added dual-notch band performance by including a parasitic resonator structure. As a result, the antenna may function well in wideband settings without interfering with WLAN and WiMAX systems. Arumugam, S., & Manoharan, S [5]. made a significant breakthrough by designing an ultra-wideband planar antenna with omnidirectional radiation and an efficiency of over 80% for the UWB spectrum. In order to increase the impedance bandwidth while preserving compactness, this design used a faulty ground structure. Additionally, Chen et al. [6] created a metasurface-based miniaturized wideband planar antenna that greatly increased gain and bandwidth while decreasing size. Their research highlights how sawtooth-inspired metasurfaces can be used to create small UWB antennas while maintaining performance.

A low-profile, wide-scanning planar antenna for UHF applications was studied by Sun et al. [7] demonstrating the viability of using sawtooth shapes in highly directive, phased array systems. This antenna is very versatile for contemporary wireless communication systems because of its remarkable bandwidth coverage and scanning capabilities throughout UHF frequencies. Metamaterials have been used in recent research to enhance antenna performance. Abdelkarim et al. [8] used complementary split-ring resonators (CSRR) to design a 5G planar antenna. By employing metamaterial superstrates, their antenna was able to boost gain from 4.66 dBi to 5.67 dBi, demonstrating the significance of negative permittivity in gain augmentation. Furthermore, Kim et al. [9] fitted a four-arm log-periodic antenna with a hybrid reflector made of inhomogeneous metamaterial components. With a 4 dBi realized gain over an impedance bandwidth of 11.6:1, the use of metamaterials improved the radiation performance. Gain and bandwidth are greatly increased by the use of metamaterials. Two decades of metamaterial-loaded planar antennas were evaluated by Esmail et al. [10] who observed that configurations containing zero-index materials (ZIM) and gradient refractive index (GRIN) metamaterials significantly increase bandwidth while preserving compact antenna size.

Syed et al. [11] created a log-periodic antenna filled with metamaterial for microwave imaging in a different study. This design demonstrated how metamaterials can improve gain and impedance matching for UWB applications by achieving a maximum gain of 5.5 dB and a bandwidth of 3 GHz (2–5 GHz). The results of using metamaterials in UWB antennas are still encouraging. A dual-layer microstrip planar antenna with a broad operational passband between 1.03 GHz and 9 GHz was proposed by Trivedi et al. [12] for radar imaging. The design of radar imaging systems, where wide bandwidth and high gain are essential, was optimized with the usage of metamaterials. Additionally, metamaterials enable substantial downsizing without sacrificing functionality. A non-resonant metamaterial structure was created by Zhai et al. [13] to increase the gain of a planar log-periodic. A metamaterial-loaded antenna that improves efficiency and gain for wideband applications was proposed by Neeshu and Tiwary [14]. Al-Hasan et al. [15] developed an electromagnetic bandgap filter inspired by metamaterials in 2023 with the goal of reducing electromagnetic interference by ultra-wide stopband screening. In [16] Govindaraj et al. [16, 17] explained about power reduction strategies. The sawtooth shape provides improved bandwidth and compactness for the log-periodic design by Yao et al. [18]. Li et al. [19] proposed a 3D printed high-temperature ceramic conformal array antenna. A Metamaterial-based Log-Periodic Antenna (LPA) for Ultra-Wideband (UWB) applications by Yadav et al. [20]. Niu et al. [21] proposed a technique in breaking the trade-off between complexity and absorbing performance in metamaterials through intelligent design. traditional UWB antennas often face challenges was discussed by Wang et al. [22]. Ren et al. [23] discussed about Ultra-Broadband Perfect Absorbers Based on Biomimetic Metamaterials with Dual Coupling Gradient Resonators. The integration of metamaterials engineered materials with properties by Zhang et al. [24]. Bai et al. [25] proposed Omnidirectional sound wave absorption based on the multi-oriented acoustic meta-materials. The log-periodic design, known for its self-similar structure by Yadav et al. [26].

Yadav et al. [27] proposed beam steered graphene-based Yagi–Uda array antenna with a transverse magnetic to hybrid mode conversion approach. metamaterial-based LPAs specifically cater to the needs of antenna by Yao et al. [28]. Sun et al. [29] discussed about a High-Precision and Fast Modeling Method for Amplifiers. Recent trends in antenna design have included reconfigurable designs and the use of emerging 2D materials. In particular, Yadav et al. proposed a beam-reconfigurable circular polarized antenna based on a benzene structure for biomedical applications. This antenna provided enhanced beam steering, polarization purity, and resolution abilities, underpinning the importance of symmetry and polarization-reconfigurable designs for biomedical imaging applications. In terahertz (THz) technology, work on the use and analysis of hybrid material and optimized solutions has surfaced as a rapidly developing area.

In work [30], a method for optimizing a THz antenna operating on SPP by means of mode mapping and a machine learning algorithm has been proposed and implemented for a hybrid material structure for a THz antenna operating on SPP modes. Analysis indicated improvements for bandwidth, impedance matching, and radiation efficiency, indicating that intelligent algorithmic techniques for electromagnetic structure design and optimization are a developing area. Graphene SPP-based reconfigurable antennas for environmental sensing purposes were explored in work [31]. Material engineering and substrate engineering have also proven to play significant roles in determining the effect of THz antennas. A dual-substrate graphene THz antenna was proposed for superior sensing tasks in [32]. By leveraging material innovations, a micro structured borophene circularly polarized slotted antenna was proposed for dual-band THz sensing and detection in [33]. The proposed design demonstrated better sensing capabilities and improved polarization performance, establishing borophene’s potential to serve as a substitute for plasmonic materials in THz antennas [34]. These efforts clearly indicate the shift in interest towards developing dynamically configurable, plasmonic, and material-optimized antennas.

### Key contributions

Metamaterials have great potential to improve bandwidth, gain, and downsizing when used into log-periodic sawtooth planar antennas for UHF-UWB applications. Because of these developments, metamaterial-based antennas are extremely versatile for use in 5G, radar, and microwave imaging, among other contemporary communication technologies. For even bigger performance gains [35], future research might investigate more intricate metamaterial constructions. This work proposes a wideband ultra-wideband (UWB) antenna that operates between 0.1 and 1.3 GHz and is intended for UWB applications. The antenna’s unique two-arm log-periodic sawtooth planar design provides a lower dimension and a higher gain than traditional dipole and bowtie antennas. This construction and design make use of metamaterial [36]. The antenna performs well in the 0.1 to 1.3 GHz range, according to the data, with a reflection coefficient (S11) of less than − 10 dB and a VSWR of less than 2.

### Organization of paper

Section 1 describes the introduction part and previous work, key contribution are illustrated in Sect. 2. Proposed method is elaborated in Sect. 3 and in Sect. 4 results and discussions are explained. Conclusion is descried in Sect. 5.

## Proposed log periodic antenna

**Fig. 1:**
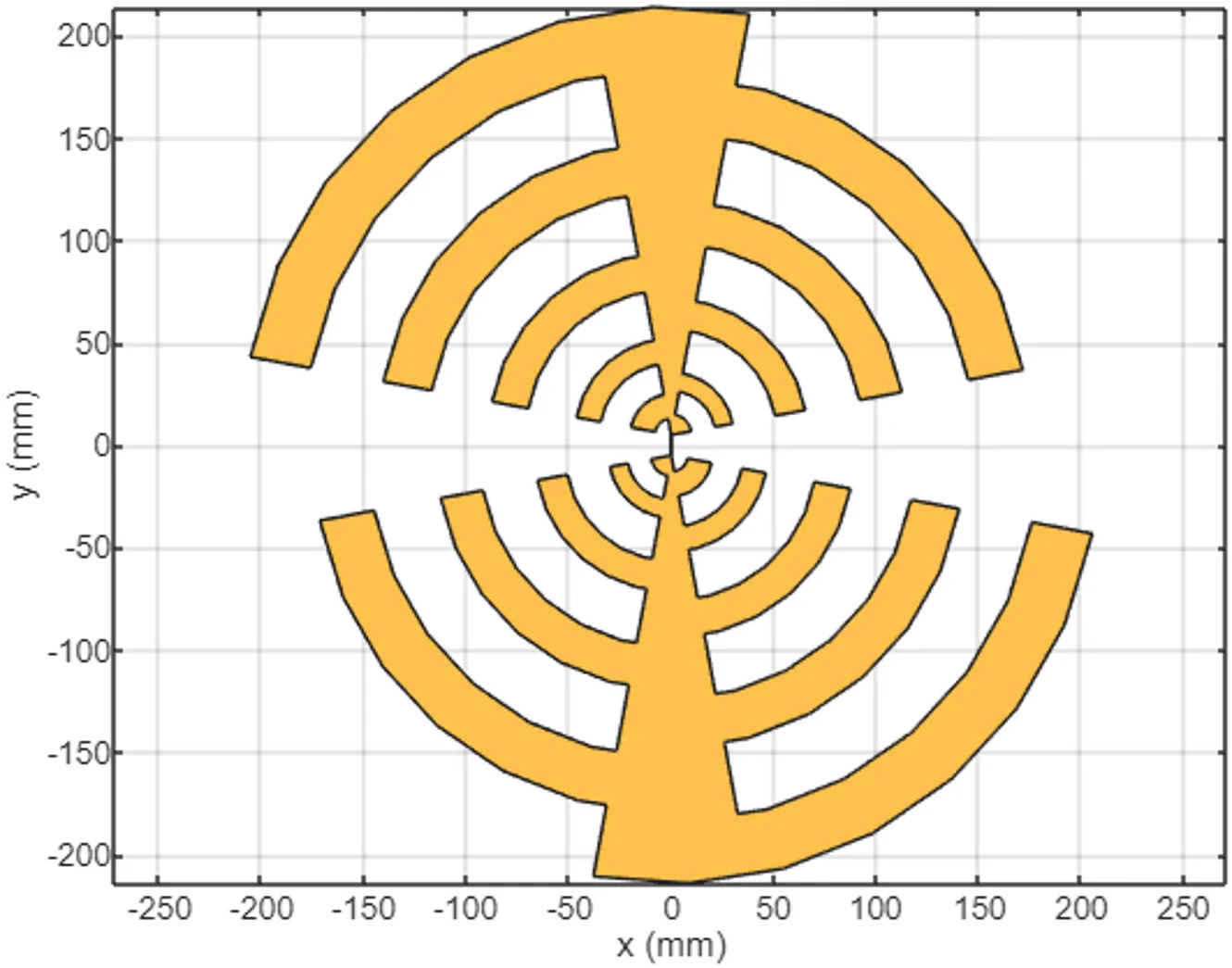
Proposed toothed log periodic Antenna Geometry

Two-arm sawtooth log-periodic for Ultra Wide Band antenna is proposed. It resembles a bowtie antenna with two arms positioned atop a metamaterial substrate. The design is based on the log-periodic antennas, dipole, spiral and bow-tie. It features two sawtooth wings that are symmetrical in the middle, much like a bow-tie antenna. The complete antenna is installed on a 400 mm diameter (Wp) square dielectric substrate, as shown in Fig. 1.

**Fig. 2:**
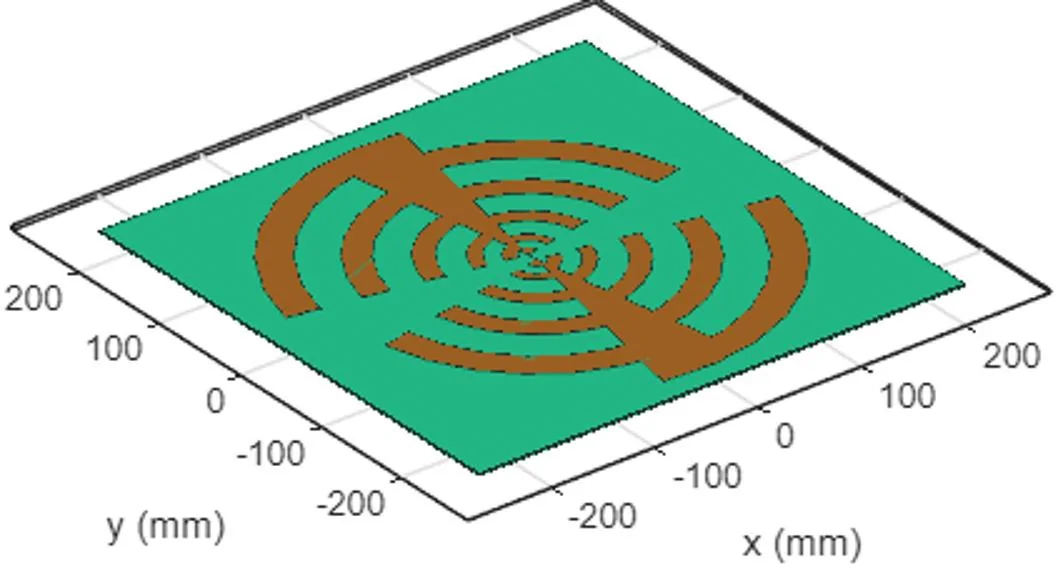
Proposed toothed log periodic with feed point

Mounted on a square metamaterial substrate, the suggested microstrip antenna has dimensions of 400 mm for the edge length (Ws), 4.8 for the dielectric constant (εr), 0.03 for the attenuation coefficient (tan δ), and 38 micrometers for thickness. As seen in Fig. 1. this antenna has a higher gain and provides broadband performance in comparison to dipole and bowtie antennas [37]. Ground Penetration Radar (GPR) systems and other UHF range applications that demand a large bandwidth are a good fit for it. With a separation distance (s) of 16 mm between the wings, the feed point is situated between the antenna’s two arms, as seen in Fig. 2.

**Fig. 3:**
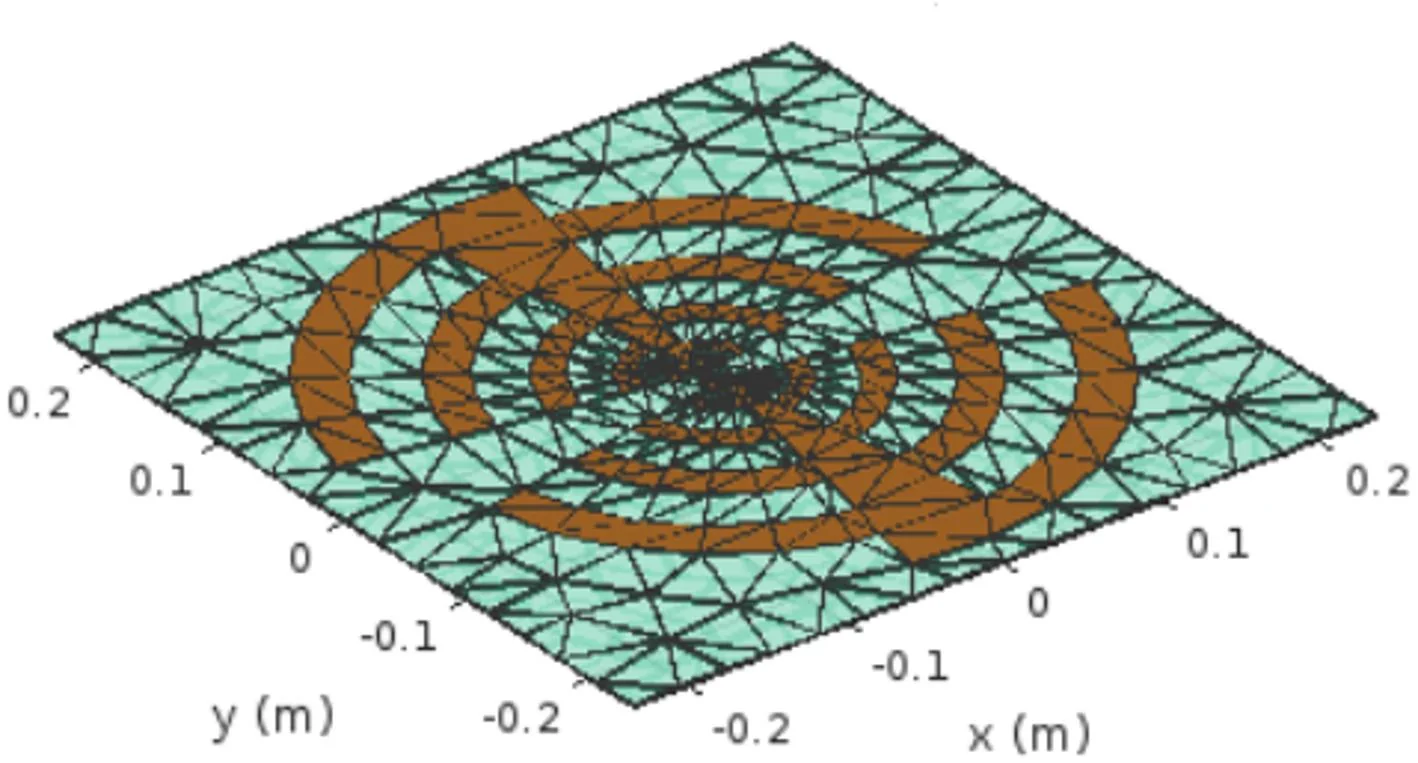
Max edge length of proposed antenna

The antenna needs to have a big aperture angle (θ) and a relatively small angle (φ) in order to attain ultra-wide bandwidth. Simulations and optimizations show that the best results are obtained with an aperture angle (θ) of 180° and an angle (φ) of 100°. As shown in Fig. 3, the antenna’s design consists of two symmetrical wings with various arcs with varied radii and slots with different widths. A sawtooth pattern is produced by the arcs’ symmetrical staggered placement along the horizontal axis. At the feed point, the centers of all arcs and slots are the same. The arc radii (r) and slot width (d) are shown in Table 1. There are nine slots in the antenna, and the biggest arc, which defines the total radius, has a radius (r10) of 200 mm. The antenna is excited using 50-Ω feed, where the graphene radiator is coupled via a metal contact pad. This approach avoids direct feeding of graphene by a conventional microstrip line and reflects practical fabrication constraints.

**Table 1:** Arc radius and slot width

*n*	Arcradius *r*_*n*_(mm)	Slotwidth d_*n*_(mm)
1	10	9
2	20	12
3	30	15
4	45	18
5	65	21
6	85	24
7	110	27
8	135	30
9	165	33
10	200	--

The ratio of successive element radii (/−1) in typical log-periodic antennas is a constant k, as is the spacing between elements (/−1) (d n ​ /d *n* − 1). The suggested antenna is not strictly log-periodic, even though it looks like traditional log-periodic designs. The radius of the elements are changed by arrangements. As a result, more elements in the same space can be supported by proposed antenna [38], which enables it to resonate and function best at lower frequencies. The conductivity of graphene is largely influenced by carrier concentration, which depends on factors like the applied electric field, temperature, and impurity scattering [39]. The General equations are illustrated from Eq. (1) to (3).

The basic equation for conductivity σ in terms of mobility µ and charge carrier density n is given by:

1 $$\:{\upsigma\:}=\mathrm{n}\mathrm{e}{\upmu\:}$$

Where elementary charge is represented by e and due to unique properties of grapheme n can be tuned by an external gate voltage. In monolayer graphene, the carrier density n is proportional to the gate voltage Vg relative to the Dirac point and the capacitance Cg per unit area of the gate dielectric

2 $$\:\mathrm{n}=\mathrm{Cg}/\mathrm{e}\left(\mathrm{V}\mathrm{g}-\mathrm{V}\mathrm{D}\mathrm{i}\mathrm{r}\mathrm{a}\mathrm{c}\right).$$

where, VDirac​ is the voltage at the Dirac point.

At low temperatures, the minimum conductivity near the Dirac point is approximately.

3 $$ \sigma _{{\min }} \approx 4e^{2} /h $$

where h is Planck’s constant. This represents the minimal conductivity in graphene due to quantum mechanical effects, even when *n* ≈ 0.

Thermally activated carriers at finite temperatures TTT, the conductivity is modified by thermally generated electron-hole pairs.

4 $$\:{\upsigma\:}\left(\mathrm{T}\right)={\upsigma\:}\mathrm{m}\mathrm{i}\mathrm{n}\text{}+{\upalpha\:}\mathrm{T}$$

where α is a constant that depends on graphene’s properties and scattering mechanisms.

The equations describing graphene’s conductivity can be complex, involving quantum mechanical considerations. The grapheme conductivity can be altered for antenna applications as per the equation [17] given from (5) to (8)

5 $$\:{\upsigma\:}=\:{\upsigma\:}\mathrm{i}\mathrm{n}\mathrm{t}\mathrm{e}\mathrm{r}+{\upsigma\:}\mathrm{i}\mathrm{n}\mathrm{t}\mathrm{r}\mathrm{a}$$

6 $$\sigma {\mathrm{inter}}\left( {\gamma ,\omega ,~\mu c,T} \right) = - je24\pi h~\ln (2\left| {\mu c} \right| - \left( {\omega - j2\gamma } \right)/h2\left| {\mu c} \right| + \left( {\omega - j2\gamma } \right)h $$

where e is the electron charge, kB is the Boltzmann constant, γ is the scattering rate, *ℏ* is the reduced Planck’s constant, µc is the chemical potential (eV), T is the mean temperature (K), and ω is the frequency in rad/s. The chemical potential µc is determined by the carrier density n (m-2).

7 $$ \sigma {\mathrm{intra}}\left( {\gamma ,\omega ,~\mu c,T} \right) = \frac{{\pi \hbar 2e2kBT\tau \left[ {kBT\mu c + 2\ln \left( {e - kBT\mu c + 1} \right)} \right]1}}{\omega } - j2\gamma $$

8 $$\:\mu\:\mathrm{c}={\upnu\:}\mathrm{f}\:{\hbar}\sqrt{\mathrm{n}}{\uppi\:}$$

The response of graphene’s inter band conductivity [39] is frequency-dependent, making it suitable for antenna applications where different frequencies are of interest. The Fermi level, µc can be controlled by applying a gate voltage. This tunability is one of the key advantages of using graphene in antennas, as it enables reconfigurable antenna behavior. Scattering Rate for the carrier relaxation rate, impacting the width of the conductivity response [40]. Higher scattering rates correspond to shorter relaxation times and influence the conductivity’s imaginary part [41].

Planar antennas where effective refractive indices [42] and surface conductivity play roles in the dispersion relation equation from (9) to (11) .

9 $$ \sqrt n ^{2} - n2eff + n\sqrt n ^{2} - n2eff + ~4\pi c\sigma w\sqrt 1 - n2eff\sqrt n ^{2} - n2eff = 0$$

Where.

n: This usually denotes the medium’s refractive index, through which the wave is traveling.

The effective refractive index, or neff, is a measurement that takes into consideration how the materials and structural geometry interact to affect the way waves travel [43].

c: In a vacuum, this is the speed of light.

σ Surface conductivity of graphene (or another conductive material), which is often frequency-dependent.w: Likely represents the width of the graphene or conductive layer.

10 $$\:{\uplambda\:}\mathrm{s}\mathrm{p}\mathrm{p}=2\mathrm{*}{\uppi\:}\mathrm{R}\mathrm{e}\left(\mathrm{k}\mathrm{s}\mathrm{p}\mathrm{p}\right)$$

11 $$\:\mathrm{k}\mathrm{S}\mathrm{P}\mathrm{P}=\:\mathrm{k}0\left(\mathrm{n}\mathrm{e}\mathrm{f}\mathrm{f}\right)$$

neff​ represents the complex effective index of the surface plasmon polariton (SPP) modes. k0 = 2πλ0/k0 ​ is the free-space wavenumber, where λ0​ is the wavelength in free space. The resonant length L1​ is defined as half of the surface plasmon [44] polariton wavelength, λSPP and L1 = λSPP/2 This setup relates the free-space properties to the confined SPP modes, where neff​ captures the propagation characteristics of the SPP wave along the interface, and L1​ defines the resonance condition for the SPP mode [45].

**Fig. 4:**
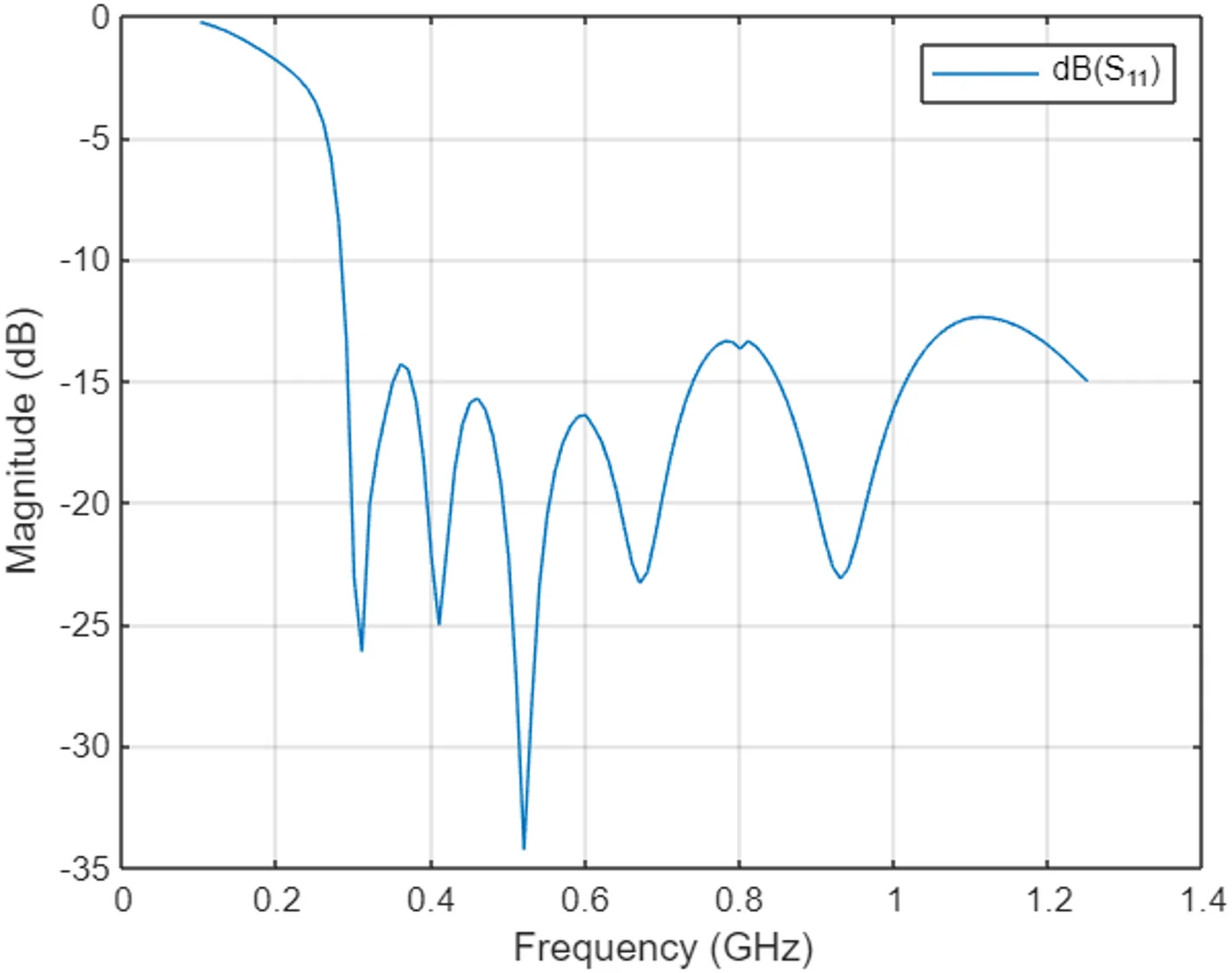
S11 Reflection coefficient

The reflection coefficient, or S11, indicates how much power is reflected back from the input port of an antenna or radio frequency circuit [46]. It gauges the impedance match between the antenna and the source or transmission line. Better impedance matching is shown by a lower S11 value, which means that the antenna receives the majority of the power from the source [47]. Proposed approach provides superior impedance matching, as evidenced by the reflection coefficient [48], which is typically less than 10dB. The antenna is excited using a CPW-based 50-Ω feed, where the graphene radiator is coupled via a metal contact pad. This approach avoids direct feeding of graphene by a conventional microstrip line and reflects practical fabrication constraints.

**Fig. 5:**
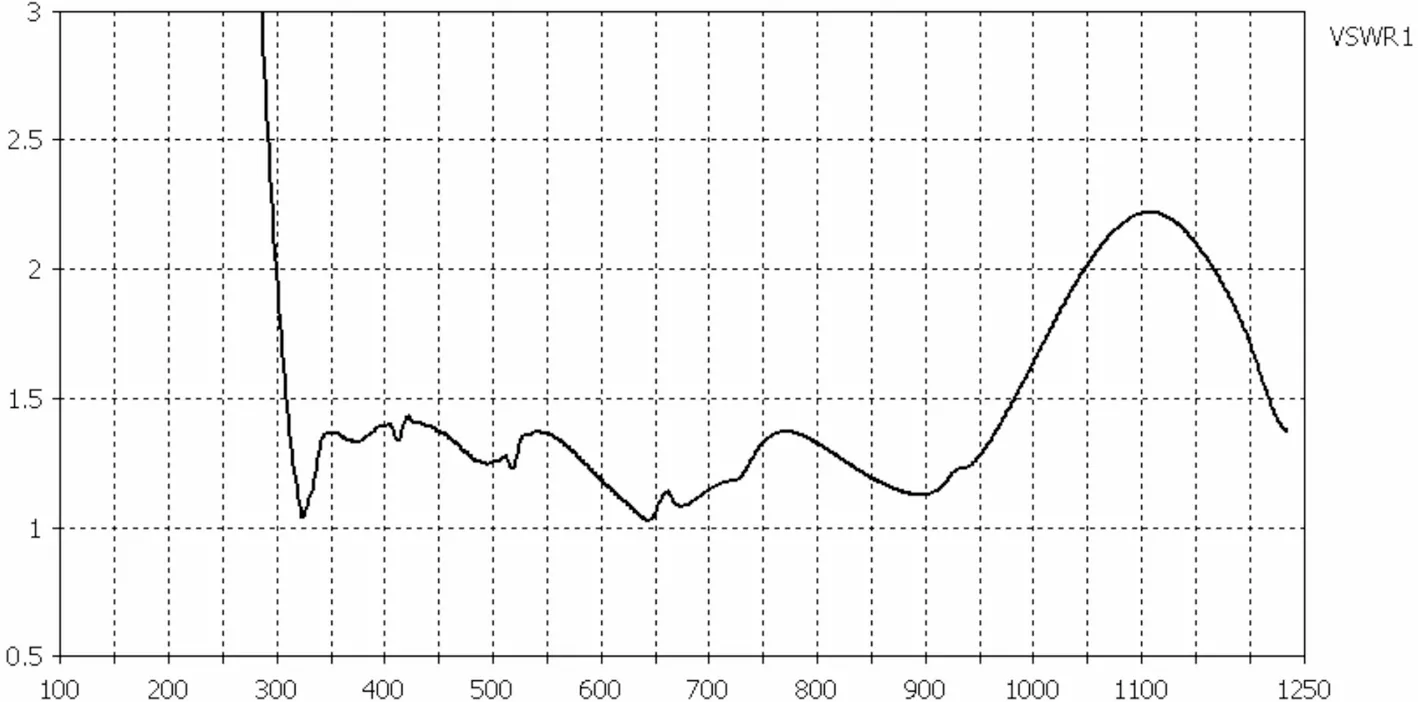
SWR of proposed method

The degree of mismatch between an antenna [49] and the transmission line or source is indicated by the Voltage Standing Wave Ratio (VSWR). It is directly correlated with the reflection coefficient S11 and measures the efficiency of RF power transmission from the source to the load (in this case, the antenna). Lower VSWR means more efficient power transfer [50]. A high VSWR value leads to increased reflected power, which can result in signal loss, increased heat, and potential damage to the transmitter. In general, VSWR value 1.0 to 1.5 indicates excellent match and very little reflection and VSWR between 1.5 and 2.0 shows a good match; acceptable in most applications. Proposed antenna has VSWR on an average between 1 and 1.2.

## Experimental results

**Fig. 6:**
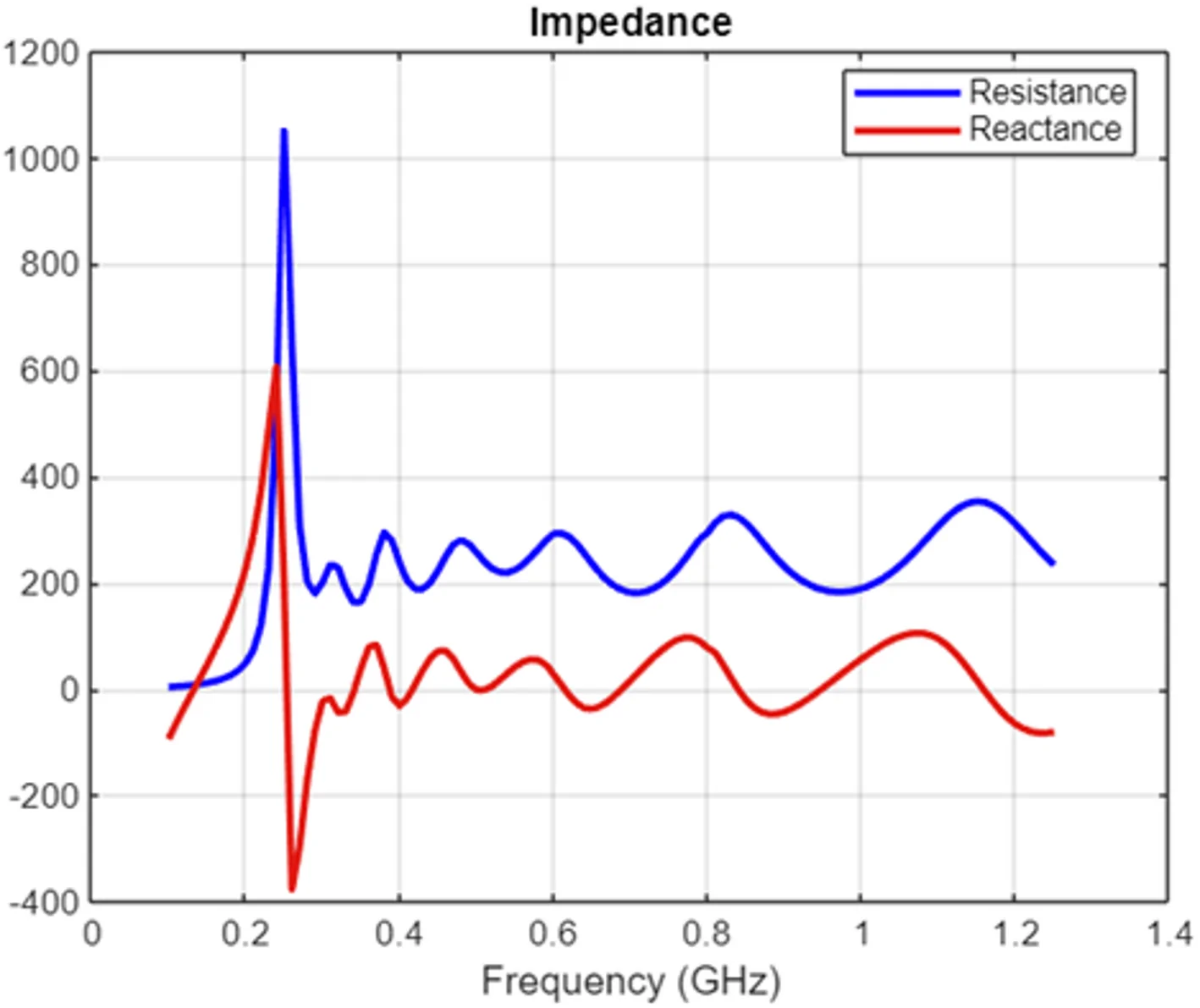
Impedance plot of proposed Antenna

**Fig. 7:**
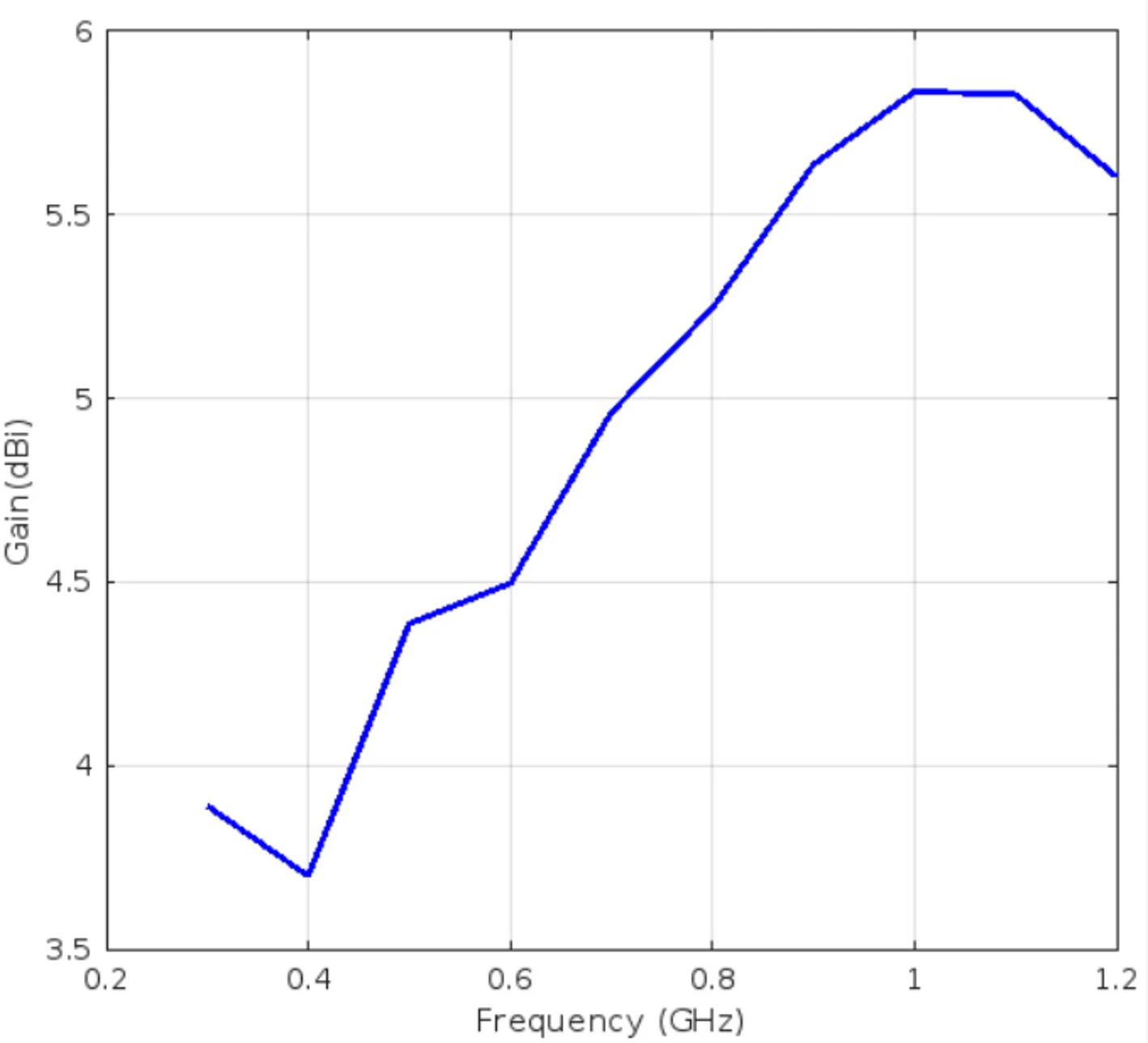
Gain of proposed Antenna

**Fig. 8:**
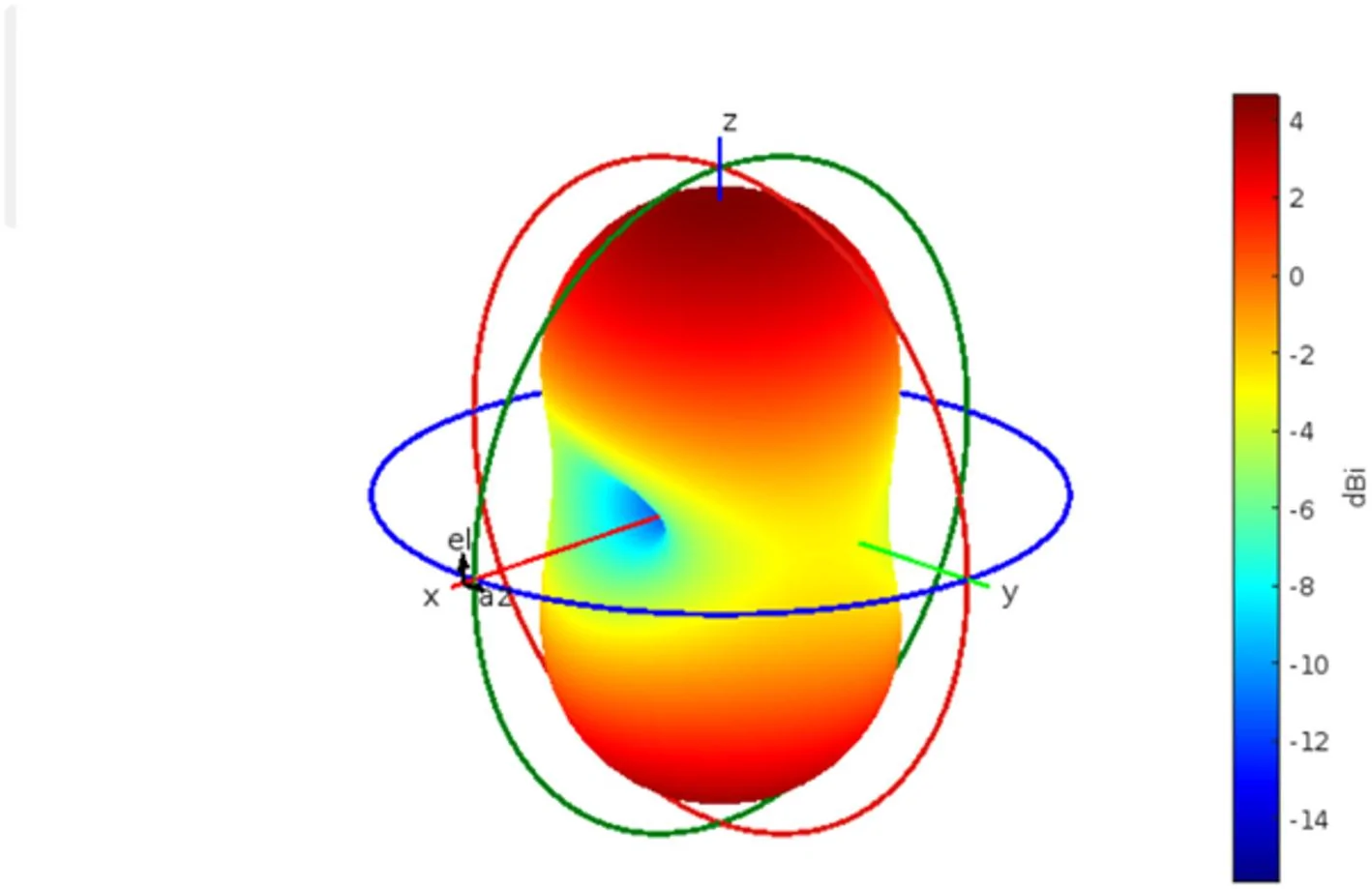
Radiation pattern of proposed Antenna

**Fig. 9:**
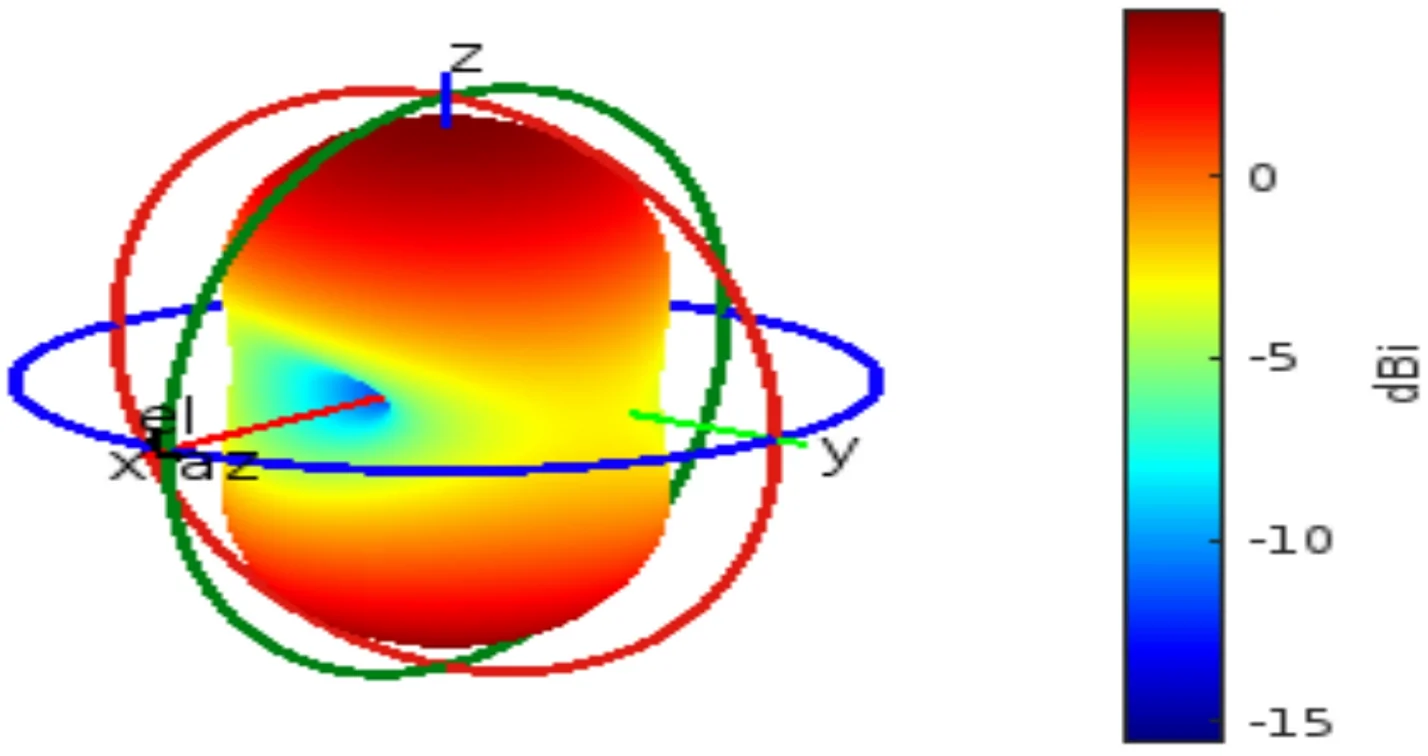
Radiation pattern of proposed Antenna

Figure 4 displays the simulation results for the antenna’s return loss parameter (S11). S11 stays below − 10 dB in the frequency range of 0.1 GHz to 1.3 GHz, where the proposed antenna resonates. Similarly, as illustrated in Fig. 5, the standing wave ratio (VSWR) for the frequency range of 300 MHz to 1.3 GHz is 1dB. Figure 6 shows the real part of the impedance plot of the proposed antenna. Figure 7 displays the antenna gain graph throughout the operational frequency range [51]. It shows that during the frequency range of 300 MHz to 1000 MHz; the gain gradually rises from 3.8 dB to 6 dB. Figures 8 and 9 shows the proposed antenna’s 3D radiation patterns at 600 MHz Because of its two-way focus, the radiation pattern is perfect for applications that need high-gain performance, long-distance communication, and a low beamwidth.The antenna achieves a gain of 6 dB at 600 MHz.

Resistance is a part of impedance that represents either radiated power or power lost as heat, while impedance includes both resistance and reactance. Together, they define the antenna’s ability to efficiently radiate power and match with the transmission line. Achieving impedance and resistance match in an antenna is essential for maximizing efficiency, reducing reflections, and ensuring that the power provided to the antenna is effectively radiated as electromagnetic waves. In proposed antenna the resistance and impedance match are very close is shown in Fig. 6.

The gain of an antenna shown in Fig. 7 measures its effectiveness in concentrating radiated power in a specific direction, making it a crucial parameter for applications requiring focused, long-range signal transmission. High gain improves signal strength and range, but often requires a trade-off with beamwidth and antenna size.

The radiation pattern shown in Fig. 9 which is a fundamental characteristic of an antenna, determining its coverage, directionality, and suitability for specific applications. It’s essential for designing and optimizing antenna systems for maximum performance and minimal interference.

**Table 2:** Performance comparison of proposed and existing antenna

S. No	Reflection Coefficient S11		VSWR		Gain in dB	
	Existing Geethamani et al. 2020 [18]	Proposed	Existing Geethamani et al. 2020 [18]	Proposed	Existing Geethamani et al. 2020 [18]	Proposed
1	− 26 dB	10 dB	1.3	1–1.5	3.23	6

A performance comparison between the proposed and existing antennas confirms the design’s superior effectiveness. Table 2 summarizes these improvements, specifically contrasting the current work with Geethamani et al. (2020). Among the critical parameters evaluated, the reflection coefficient S_11_ is paramount, as it quantifies the power loss resulting from impedance mismatch. In the proposed antenna, is an indicator of an excellent matching condition and minimum reflected power. Secondly, the Voltage Standing Wave Ratio (VSWR) also verifies the effectiveness of the enhancement made. It is evident that the VSWR of the proposed antenna ranges from 1 to 1.5, which is near to the perfect result of 1. Ideally, when VSWR is 1, it implies that the network has zero standing waves. Even though the VSWR of the already-existing design is 1.3, the VSWR of the proposed design is constantly low within the frequency band. Finally, the gain performance results indicate a large improvement in the proposed antenna. The value of the gain has increased from 3.23 dB in the existing work to 6 dB in the proposed antenna, which is a major improvement and confirms the better radiating efficiency and purity of high directivity, suitable for long-distance communication systems. Thus, from the detailed comparison, it can be seen that the proposed antenna has definitely outshined its existing counterpart in impedance matching, minimizing reflections, and radiation efficiency, and it has proven the effectiveness of modifications to achieve better antenna performance.

## Conclusion

In the proposed method, a graphene-based antenna with an S11 -10 dB gain was constructed for the UWB working frequency range (0.1–1.3 GHz). The real antenna has teeth based on graphemes and is a log periodic antenna. The simulation results were achieved by changing the chemical potential of the graphene material. The suggested antenna’s maximum bandwidth falls between 300 and 1.3 GHz. Additionally, it features a 6 dB boost for frequencies between 0.1 and 1.3 GHz. The results of the measurements show a good match between the simulation and the implementation. Based on the findings, the recommended antenna can be used suitably for long-distance broadband UWB applications, such as GPR systems.

## Data Availability

The datasets used and/or analysed during the current study available from the corresponding author on reasonable request.

## Code availability

The relevant code with the manuscript is also available and would be available, if will be asked to do so later.
